# Transcription Factors in the Development and Function of Group 2 Innate Lymphoid Cells

**DOI:** 10.3390/ijms20061377

**Published:** 2019-03-19

**Authors:** Takashi Ebihara, Ichiro Taniuchi

**Affiliations:** Laboratory for Transcriptional Regulation, RIKEN Center for Integrative Medical Sciences (IMS), 1-7-22 Suehiro-cho, Tsurumi-ku, Yokohama 230-0045, Japan; ichito.taniuchi@riken.jp

**Keywords:** group 2 innate lymphoid cells, transcription factor, exhausted-like ILC2, Runx

## Abstract

Group 2 innate lymphoid cells (ILC2s) are tissue-resident cells and are a major source of innate T_H_2 cytokine secretion upon allergen exposure or parasitic-worm infection. Accumulating studies have revealed that transcription factors, including GATA-3, Bcl11b, Gfi1, RORα, and Ets-1, play a role in ILC2 differentiation. Recent reports have further revealed that the characteristics and functions of ILC2 are influenced by the physiological state of the tissues. Specifically, the type of inflammation strongly affects the ILC2 phenotype in tissues. Inhibitory ILC2s, memory-like ILC2s, and ex-ILC2s with ILC1 features acquire their characteristic properties following exposure to their specific inflammatory environment. We have recently reported a new ILC2 population, designated as exhausted-like ILC2s, which emerges after a severe allergic inflammation. Exhausted-like ILC2s are featured with low reactivity and high expression of inhibitory receptors. Therefore, for a more comprehensive understanding of ILC2 function and differentiation, we review the recent knowledge of transcriptional regulation of ILC2 differentiation and discuss the roles of the Runx transcription factor in controlling the emergence of exhausted-like ILC2s. The concept of exhausted-like ILC2s sheds a light on a new aspect of ILC2 biology in allergic diseases.

## 1. Introduction

Innate lymphoid cells (ILCs) are lymphocytes responsible for innate cytotoxicity or helper functions [1,2,3]. While ILCs with cytotoxic capability are conventional natural killer (NK) cells which do not have tissue-resident properties, ILCs with helper functions generally reside in the tissue, provoke immune responses against pathogens, and maintain mucosal integrity [4,5]. Helper ILCs are unable to directly sense pathogenic antigens because they do not express antigen-specific receptors such as T cell receptor and B cell receptor. Instead, helper ILCs are generally stimulated by cytokines released from damaged-epithelial cells, phagocytes, and dendritic cells [6,7,8,9,10,11]. Based on cytokine production and requirements of transcription factors, helper ILCs are classified into three distinct subsets; ILC1s, ILC2s, and ILC3s which mirror T_H_1, T_H_2, and T_H_17, respectively [3,12]. ILC1s secrete interferon g (IFNγ) and require T-bet for their differentiation [13]. T-bet is also important for the final maturation of conventional NK cells, which is highly dependent also on eomesodermin (Eomes) [14]. ILC2s are characterized by the production of the T_H_2 cytokines interleukin 4 (IL-4), IL-5, IL-13, and granulocyte-macrophage colony-stimulating factor (GM-CSF). All of these expressions are positively regulated by the transcription factor GATA binding protein 3 (GATA-3) [15]. Both RAR-related orphan receptor gamma t (RORγt) and aryl hydrocarbon receptor (AhR) are required for the differentiation of ILC3s as well as for the production of IL-17 or IL-22 by ILC3s [16,17,18]. A substantial number of ILC1s are found in the liver, intestinal intraepithelial layer, intestinal lamina propria, adipose tissue, and the skin [13,19,20,21]. ILC2s are preferentially localized in the lung, skin, adipose tissue, fat-associated lymphoid clusters, and the intestinal lamina propria [7,8,11,22]. ILC3s are abundant in the intestinal lamina propria, skin, and secondary lymphoid organs, such as Peyer’s patches and mesenteric lymph nodes [16,18,23].

All ILC subsets develop from common lymphoid progenitor cells (CLPs) [24]. During the first commitment process from CLPs to the ILC lineage, early innate lymphoid progenitor cells (EILPs) are generated [25]. EILPs give rise to conventional NK cells and all helper ILC subsets. EILPs are marked by the expression of T cell factor 1 (TCF-1), thymocyte selection associated high mobility group box (Tox), and nuclear factor, interleukin 3 regulated (NFIL3) [25,26,27]. Progenitors specific to helper ILCs are Id2^+^ common helper innate lymphoid progenitors (CHILPs) that lose the developmental potential for conventional NK cells [13]. Half of all CHILPs express promyelocytic leukemia zinc finger (PLZF) and are designated as innate lymphoid cell precursors (ILCPs) [10,24]. ILCPs can differentiate into helper ILCs except for a subpopulation of ILC3s, lymphoid tissue inducer-like (Lti-like) cells.

ILC2s have been extensively studied in the research field of allergy or parasitic worm infection [9]. Physiological roles of ILC2s are also implicated in metabolic homeostasis and virus infection [22,28,29]. Steady state ILC2s in tissues constitutively produce IL-5, which recruits eosinophils to the tissues [30]. Eosinophils are one of the essential components of allergy, as well as cytotoxic effector cells against parasitic worms. When mucosal tissues are injured by allergens or worms, IL-33, IL-25, and thymic stromal lymphopoietin (TSLP) are released from the damaged epithelial cells [31]. ILCs sense these signal cytokines by their cognate receptors and produce a variety of T_H_2 cytokines, including IL-5, IL-9, and IL-13. IL-9 is necessary for the maintenance of ILC2 activity in an autocrine manner during the early phase of an allergic reaction [32]. IL-13 from ILC2s induces chemokine CCL17 expression from dendritic cells to attract T_H_2 cells, resulting in enhanced T_H_2 responses [33]. ILC2s are an early source of amphiregulin, which is a ligand of the epidermal growth factor receptor and plays a critical role in the repair of damaged epithelial cells [28]. Therefore, ILC2s are involved in the initiation of allergy as well as tissue-repair.

The functional state of ILC2s is influenced by environmental cues. ILC2s acquire ILC1-like properties and produce IFNγ when mice are infected with viruses and bacteria or are exposed to cigarette smoke to induce chronic obstructive pulmonary-like disease [34,35]. IL-1b, IL-12, and IL-18 are involved in the phenotypic conversion from ILC2s to ILC1-like cells. ILC2s also acquire “memory-like” signatures [36]. Once ILC2s are stimulated with IL-33, the activated ILC2s live long in the tissue and produce increased levels of T_H_2 cytokines upon a second challenge. Systemic IL-25 treatment and helminth infection induce inflammatory ILC2s (iILC2s), which are characterized by IL-17 production [37]. iILC2s migrate from the site of inflammation to the systemic tissues through lymphatics in an S1P-depenent manner [38]. Despite such migratory capacity of iILC2s, the majority of ILC2s reside in the tissue during and after helminth infection [4].

Transcription factors specifically required for ILC2 differentiation have been studied. Previous studies demonstrated the importance of transcription factors, such as GATA-3, B-Cell lymphoma/leukaemia 11B (Bcl11b), growth factor independent 1 transcription repressor (Gfi-1), RAR related orphan receptor A (RORα), and v-ets erythroblastosis virus E26 oncogene homolog 1 (Ets-1), for ILC2 differentiation [7,15,39,40,41,42,43,44,45,46]. ILC2s change their characteristics in response to environments. The mechanism by which transcription factor networks dictate phenotypical changes of ILC2s remains to be determined. For a comprehensive understanding of physiological roles of ILC2s in the allergic pathology, we summarize the current understanding of the transcriptional control of ILC2 differentiation and function, followed by our recent findings regarding how Runt-related transcription factor (Runx) proteins regulate the functional state of ILC2.

## 2. Transcription Factors Involved in the Development of Group 2 Innate Lymphoid Cells (ILC2s)

### 2.1. GATA-3

**Figure 1:**
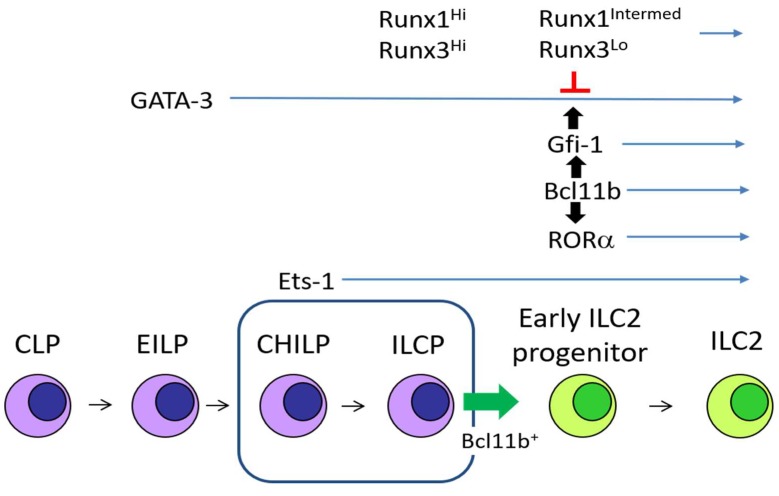
Group 2 innate lymphoid cells (ILC2) differentiation and transcription factors. GATA-3 is expressed in early innate lymphoid progenitor cell (EILP), and its expression is maintained in all ILC subsets. Ets-1 up-regulates Id2 in common helper innate lymphoid progenitors (CHILP). Bcl11b-expressing progenitors in the CHILP population give rise to ILC2 (green blocked arrow). Bcl11b positively regulates RORα and Gfi-1 both of which are indispensable for ILC2 differentiation (black blocked arrow from Bcl11b). Gfi-1 induces GATA-3 for ILC2 specification (black blocked arrow from Gfi-1). Runx proteins repress GATA-3 activity by antagonistic binding (red T-bar).

GATA-3 is necessary for the differentiation of CHILPs, which specifically give rise to all helper ILC subsets (Figure 1) [13]. While ILC1s and ILC3s express GATA-3, the expression level of GATA-3 in ILC2s is significantly higher compared to other ILCs. GATA-3 binds the gene loci which are critical for ILC2 activity. The genes positively regulated by GATA-3 include *Il5*, *Il13*, *Areg* encoding amphiregulin, *Il1rl1* encoding IL-33 receptor ST2, *Il9*, and *Il2ra* [15]. The conditional deletion of GATA-3 in mature ILC2s leads to reduced cytokine production and hypo proliferation [15,39,40]. Therefore, GATA-3 governs ILC2 identity, differentiation, homeostasis, and function.

### 2.2. Bcl11b

Bcl11b is the earliest marker for the ILC2-lineage. The Bcl11b-expressing cells among the CHILP population specifically develop into ILC2s (Figure 1) [41]. Bcl11b ablation in hematopoietic stem cells impairs ILC2 differentiation in the bone marrow and peripheral tissues. Bcl11b expression in ILC subsets is controversial. While Yu et al. suggested that Bcl11b expression among ILC subsets was restricted to ILC2s [43], and Walker et al. showed that ILC1s and ILC3s expressed Bcl11b [42]. Conditional deletion of Bcl11b in ILC2s results in the generation of ILC2s with ILC3 gene signatures [41]. Thus, Bcl11b-deficient ILC2s have a reduction in GATA-3, Gfi-1, and RORα expression, and an increase in sex determining region Y-box 4 (Sox4), RORγt, and AhR expression. Bcl11b is associated with the *Gfi1* and *Ahr* gene loci, but not the *Gata3* or *Rorc* gene loci, suggesting that Gfi1 and AhR expression may be directly regulated by Bcl11b. Enhanced Gfi-1 expression in Bcl11b-deficient ILC2s restores GATA-3 expression and down-regulates RORγt expression. Therefore, Bcl11b positively regulates GATA-3 expression and negatively regulates RORγt expression through Gfi-1 induction.

### 2.3. Gfi-1

Gfi-1 expression in ILC2 is relatively higher than other ILCs. Gfi-1 is required for ILC2 differentiation and optimal T_H_2 cytokine responses to IL-33 [44]. Gfi-1 positively regulates the expression of *Gata3* and *Il1rl1* (Figure 1). Deletion of Gfi-1 causes aberrant IL-17a production by ILC2s due to dysregulated expression of RORγt [44]. Increased RORγt expression in Gfi-1-deficient ILC2s may be explained by increased expression of Sox4, which is known to induce RORγt expression. Thus, Gfi-1 suppresses the machinery for the ILC3 lineage and maintains the effector state of ILC2s in allergic diseases.

### 2.4. RORα

Although RORα is expressed in CHILPs and all ILC subsets, it is specifically necessary for ILC2 differentiation among ILC subsets (Figure 1) [7,45,47]. A RORα-dependent ILC2 differentiation mechanism has not been described yet. Differential interaction of RORα with the gene loci in ILC2s and other ILCs should be examined to understand RORα function in ILC2 differentiation and function. RORα-deficient staggerer mice have been used to observe physiological effects resulting from ILC2 deficiency [7,45]. However, deletion of RORα dampens regulatory T cell (Treg) and ILC3 function, but not their differentiation [48,49]. These data suggest that there might be other unknown immunological defects in the RORα-deficient staggerer mice and the related mice with conditional deletion of RORα.

### 2.5. Ets1

Ets-1 induces Id2 which is critical for differentiation of ILC progenitors, CHILPs [46]. However, for unknown reasons, ILC2s without Ets-1 are reduced only in the bone marrow, but not in the lung. Ets-1 is required for ILC2s to expand and produce IL-5 and IL-13 in response to IL-33. In contrast, IL-6 and IL-9 production are up-regulated in Ets-1-deficient ILC2s cultured with IL-33. It remains to be elucidated how Ets-1 regulates the ILC2 fitness and function.

## 3. Runx Proteins and Immune Cells

### 3.1. Global Effects of Runx Proteins in Immune Cells

Runx proteins are a family of transcription factors necessary for many biological processes ranging from differentiation, function, proliferation, tumorigenesis, and cellular identity [50,51]. There are three mammal members in the Runx protein family: Runx1, Runx2, and Runx3. All Runx proteins require heterodimer formation with Subunit b of core binding factor (Cbfβ) to exert their function as transcription factors. All three Runx genes generate two transcripts from distal (P1) and proximal (P2) promoters [52]. These two transcripts generate Runx protein isoforms with distinct N-terminal sequences. Utilization of the promoters differs among cell types. *Runx1* and *Runx3* genes are expressed in many types of immune cells [50]. Runx1 is required for the emergence of hematopoietic stem cells from hemogenic endothelium. Loss of Runx1 causes embryonic lethality due to bleeding in the brain caused by the absence of platelets [53,54]. Runx1 is also involved in the early thymocyte differentiation and development of invariant natural killer T (iNKT) cells, regulatory T cells and T_H_17 cells [51,55,56,57,58]. Runx3 is a critical transcription factor for the differentiation of CD8^+^ T cells and T_H_1 cells [56,59]. Runx3 is crucial to induce effector genes such as *Ifng*, *Gzmb*, *Eomes*, and to repress *Il4* gene in T_H_1 cells. *Runx3* transcript from the P1 promoter is correlated with protein expression level in T cells because the P2-*Runx3* transcript utilizes IRES-mediated translation [52,60]. Runx2 is required for the differentiation of osteoblasts and bone formation [61,62]. Runx2 expression in hematopoietic cells is rather restricted to plasmacytoid dendritic cells [63]. Runx2-deficient plasmacytoid dendritic cells have deficits in migrating to the peripheral tissues.

### 3.2. Runx3 Is Required for the Differentiation of ILC1s and ILC3s

Helper ILCs and conventional NK cells preferentially express *Runx3* mRNA among all Runx family genes [64,65]. However, ILC subsets are characterized by the levels of *Runx3* P1 transcripts and Runx3 protein expression. High Runx3 protein expression is observed in NK cells, which are innate counterparts of CD8^+^T cells, and in ILC1s, innate counterparts of T_H_1 cells. ILC3s, innate counterpart for T_H_17 cells, express an intermediate level of Runx3, while Runx1 is likely to be a major Runx protein to drive T_H_17 program. Runx3 expression is very low in ILC2s, innate lymphoid cells. Ablation of Runx3 in all hematopoietic cells leads to a reduction of NK cells, ILC1s, and ILC3s but not ILC2s. Runx3 is necessary for optimal IFNγ production by ILC1s. Ablation of Cbfβ or a combined loss of both Runx1 and Runx3 result in a dramatic reduction of NK cells and ILC1s, suggesting that Runx1 compensates for the loss of Runx3 in these cells. ILC1s fall into an apoptotic state in the absence of Runx proteins, which is a result of low expression of anti-apoptotic Bcl2. For the development of ILC3s, Runx3 induces expression of an ILC3 master regulator gene, RORγt mainly by direct binding to the intronic enhancer region of the RORγt [66]. AhR is also required for full production of IL-17 and IL-22 together with RORγt [17]. Runx3 deletion impairs expression of *Ahr* in ILC3s. Upon a half dosage of RORγt expression caused by heterozygous gene knock-out, *Ahr* expression is reduced by half in the ILC3s. Thus, Runx3 regulates RORγt expression and its downstream target, AhR.

Lymphoid tissue-inducer cells (Lti) cells are indispensable for secondary lymphoid organogenesis and are considered as a subpopulation of ILC3s because Lti cells are characterized by and are dependent on RORγt expression. Lti cells appear in the embryonic intestine at embryonic day 12. An early study clarified the importance of Runx1 for Lti cell differentiation via RORγt induction in the embryos [67]. Although RORγt expression in Lti-cells is reduced by Runx3 deficiency, the number of Lti cells in the embryonic intestine is normal in the absence of Runx3 [64]. These data suggest that there might be distinct functions of Runx proteins for the generation of Lti cells.

Runx proteins also regulate early ILC differentiation. Runx1 and Runx3 expression in ILC progenitors increases as CLPs give rise to CHILPs and ILCPs (Figure 1) [64]. Expression of Runx1 and Runx3 reaches the highest level at an ILCP stage in the course of ILC differentiation from CLPs to ILC subsets. Runx proteins are essential to induce PLZF expression, which is the marker for ILCPs, by direct interaction with an enhancer of the *Zbtb16* locus encoding PLZF [68]. ILC1s and ILC3s down-regulate Runx1 expression but maintain intermediate to high levels of Runx3 expression [64]. In contrast, down-regulation of Runx3 occurs in ILC2 progenitor cells and ILC2s which express more Runx1 than other ILCs. Therefore, the levels of Runx3 expression specifies ILC lineages.

## 4. The Function of Runx Proteins in ILC2s

### 4.1. Runx Proteins Prevent Steady-State ILC2s from Overactivation

Runx3 deletion is not enough for obvious phenotypic changes in ILC2s [64,69]. ILC2s lacking Runx3 normally populate the peripheral tissues and are functionally intact because Runx1 is also expressed and compensate Runx3 function in ILC2s. When the function of all Runx proteins is abrogated by ablation of Cbfβ, a binding partner of Runx proteins, in the ILC2s, the ILC2s are normally present in the tissues but exhibit an activated phenotype even in the steady-state condition. Killer cell lectin-like receptor G1 (KLRG1) is a known marker for activated ILC2s, and Thy1 is down-regulated in the ILC2s when they are stimulated by IL-25. Interestingly, loss of Runx function in ILC2s induces high KLRG1 expression and low Thy1 expression with unleashed IL-5 production. As ILC2s are a major source of IL-5, which recruits eosinophils to the lung, eosinophils in the bronchoalveolar space are increased by overproduction of IL-5 from the activated ILC2s lacking Runx function. The infiltration of eosinophils to the bronchoalveolar space seems to occur at subclinical levels because lung epithelial cells are not significantly damaged by the adoptive transfer of the Cbfβ-deficient ILC2s.

The activated ILC2 phenotype caused by Cbfβ deficiency is reminiscent of those observed in the T cells lacking Cbfβ function. T_H_2 skewing occurs in CD4 T cells in the absence of Cbfβ, resulting in asthma-like symptoms in the lung [59]. Runx/Cbfβ complexes associate and antagonize GATA-3, a master regulator of T_H_2 cells, in T cells [70]. Runx/Cbfβ complexes also suppress the expression of IL-4, which is a critical T_H_2 cytokine for type 2 immunity, through binding to a silencer region in the *Il4* gene locus [59]. Therefore, the balance between Runx and GATA-3 dictates the functional state of T_H_2 cells.

**Figure 2:**
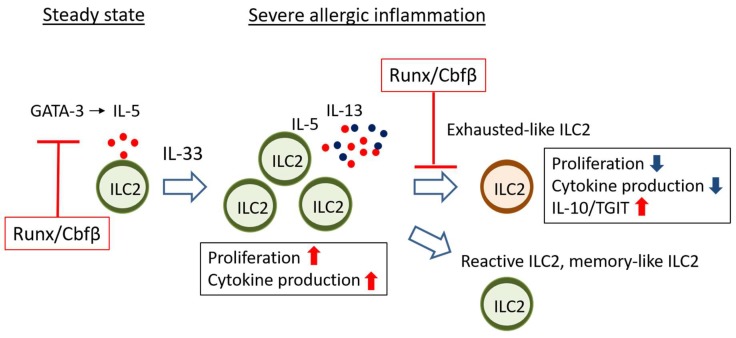
Runx/Cbfβ complexes differentially regulate ILC2 effector function at steady state and in severe inflammation. In the steady state of the lung, Runx/Cbfβ complexes suppress ILC2 activity through antagonistic binding to GATA-3 which positively regulates ILC2 effector function including constitutive IL-5 production (left red T-bar). During severe allergic airway inflammation, Runx/Cbfβ complexes are essential for ILC2s to respond to IL-33 and maintain their activity. Runx/Cbfβ complexes inhibit the emergence of exhausted-like ILC2s which are normally rare at the site of severe inflammation (right red T-bar). Red dots: IL-5. Blue dots: IL-13.

ILC2 activity at steady state is also determined by the balancing action of Runx proteins against GATA-3 (Figure 2). While GATA-3 overexpression increases IL-5 production in ILC2s, Runx3 cancels such effects in ILC2s [69]. In addition to IL-5, transcriptome analysis demonstrates that many genes regulated by GATA-3 are inversely regulated by Runx proteins. In steady-state ILC2s, IL-5 production is increased by the absence of Cbfβ, presumably via antagonizing GATA-3 function. However, we cannot deny the possibility that Runx/Cbfβ complexes work as a direct repressor for the *Il5* gene as is the case for *Il4*, because Cbfβ associates with several genomic regions around the *Il5* gene in ILC2s. Deletion of either Runx1 or Runx3 alone does not induce IL-5 overproduction from ILC2s. However, when both Runx1 and Runx3 are deleted, ILC2s show the activated phenotype, suggesting that the redundant function of Runx proteins restrains the basic activity of ILC2s in steady-state.

### 4.2. Runx Proteins Inhibit the Emergence of Exhausted-Like ILC2s during Allergic Inflammation

ILC2s are activated by IL-33, IL-25, TSLP, TNF superfamily ligand TL1A (TL1A), and neuropeptides such as vasoactive intestinal polypeptide (VIP), neuromedin U and, calcitonin gene-related peptide [6,11,30,31,71,72,73]. Furthermore, IL-33 is the most potent ILC2 stimulator in vitro and in vivo and plays an important role in the pathogenesis of allergy. Interestingly, Cbfβ-deficient ILC2s have defects in ILC2 cytokine production and proliferation during in vitro responses to IL-33 [69]. Expression of *Il5*, *Il9*, *Il13*, *Csf2* encoding GM-CSF, and *Areg* encoding amphiregulin upon IL-33 stimulation is all downregulated in Cbfβ-deficient ILC2s. In addition, the expression of many molecules critical for ILC2 activity is dampened in the Cbfβ-deficient ILC2s. ILC2s have machinery for extending immune responses upon their activation. For instance, IL-9 produced by ILC2s binds IL-9 receptor on ILC2s and stimulates their expansion [32]. Inducible costimulatory ligand (ICOSL) induced on the activated ILC2s contributes to their survival and cytokine production by receiving ICOS signals [74]. Activated ILC2s express a variety of neuropeptide receptors including Vipr2, a VIP receptor, and Nmur1, a neuromedin receptor for expansion and cytokine production of ILC2s during allergy [30,71,72,73]. Surprisingly, expression of *Il9*, *Il9r*, *Icos*, *Vipr2*, and *Nmur1* in activated ILC2 are all impaired by Cbfβ-deficiency [69]. Furthermore, ILC2s lacking Cbfβ show up-regulated expressions of a series of T-cell exhaustion marker genes, such as *Tigit*, *Il10*, *Prdm1*, *Ctla4*, and *Lag3*. It has been documented that T cells acquire a state of dysfunction, called T cell exhaustion, after chronic exposure to cancer and viruses as a result of continuous antigen stimulation [75,76,77]. Exhausted T cells are characterized by impaired ability of proliferation, cytokine production and, effector function after re-stimulation. Expression of a variety of inhibitory receptors such as programmed cell death 1 (PD-1), T cell immunoreceptor with Ig and ITIM domains (Tigit), and lymphocyte Activation Gene-3 (Lag3) are highly induced on exhausted T cells. We, therefore, define hyporesponsive ILC2s with increased expression of T cell exhaustion markers as “exhausted-like” ILC2s.

Since both Tigit and IL-10 are highly expressed in Cbfβ-deficient ILC2s, these two molecules could serve as good markers to define ILC2s with hyporesponsive characteristics. Indeed, a low number of hyporesponsive Tigit^+^IL-10^+^ ILC2s emerge at the site of severe allergic inflammation even in *wild-type* mice [69]. Severe allergy is known to induce IL-10-producing ILC2s, which are associated with decreased eosinophil recruitment in the lung [26]. Tigit expression is restricted to high IL-10-producers in the activated ILC2s [69]. The low reactive Tigit^+^IL-10^+^ ILC2s also express high levels of ILC2 activation markers such as PD-1, KLRG1, and glucocorticoid-induced tumor necrosis factor receptor (GITR). When mice are intranasally administrated with papain, a proteinase allergen, the bronchoalveolar space is the most inflamed site in the lung. The Tigit^+^IL-10^+^ ILC2s with low reactivity can be found only in the bronchoalveolar space, but not in the lung during severe subacute allergy induced by high doses of papain. The absence of Cbfβ in ILC2s enhances the generation of the Tigit^+^IL-10^+^ ILC2s and ameliorates allergic inflammation induced by papain inhalation in the subacute phase. However, when mice are chronically inoculated with papain for a month, increased low reactive Tigit^+^IL-10^+^ ILC2s from Cbfβ deficiency do not reduce the degree of chronic inflammation. If mice have the papain treatment interval for recovery after the first course of challenges, the emergence of the low reactive Tigit^+^IL-10^+^ ILC2s enhanced by Cbfβ deficiency is associated with attenuation of allergic inflammation induced by second challenges of papain treatment. Therefore, the presence of exhausted-like Tigit^+^IL-10^+^ ILC2s is likely to reduce acute deterioration of chronic allergy.

**Figure 3:**
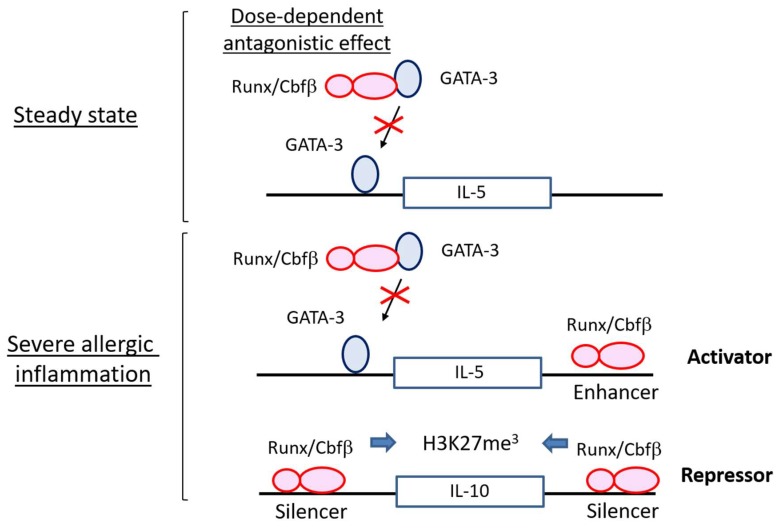
Transcriptional regulation by Runx/Cbfβ complexes in ILC2s. Runx/Cbfβ complexes antagonize GATA-3 (red cross) and suppress the ILC2 activity irrespective of allergic inflammation in a dose dependent manner. The presence of Runx/Cbfβ complexes is indispensable for ILC2s not to acquire a gene expression profile of exhausted-like ILC2s. Runx/Cbfβ complexes function as activators for gene expression of ILC2 cytokines and receptors, and as repressors for inhibitory molecules, such as IL-10.

Our data clearly show that pre-activated Cbfβ-deficient ILC2s are hyporesponsive to IL-33 stimulation in vitro and in vivo [69]. If enhanced GATA-3 activation by the absence of Cbfβ is involved in the generation of exhausted-like ILC2s, GATA-3 overexpression alone would lead to the emergence of exhausted-like ILC2s. However, ectopic expression of GATA-3 conversely elicits increased ILC2 reactivity upon IL-33 stimulation in vitro and in vivo. Of note, Runx3 overexpression over GATA-3 expression down-modulates ILC2 responsiveness, suggesting that the reactivity of ILC2s to IL33 stimulation is regulated by the balance between GATA-3 and Runx3 even in the activation phase (Figure 3). Conversely, transcription factors regulate gene expression through association with regulatory genomic regions such as promoters, enhancers, or repressors. Chromatin immunoprecipitation sequencing data suggest that the Cbfβ binding pattern in ILC2s activated with IL-33 differs from that in ILC2s without IL-33 stimulation. Interestingly, Cbfβ-binding peaks induced specifically upon IL-33 stimulation are enriched in regions nearby the signature genes for exhausted-like ILC2s. For example, the *Il5*, *Il13*, *Nmur1*, *Vipr2*, *Il10*, *Tigit*, *Prdm1*, *Lag3*, and *Ctla4* gene loci are marked by Cbfβ-bindings specific to IL-33 stimulation. These Cbfβ-binding peaks are not bound by GATA-3, suggesting that regulation of the activity of those possible regulatory regions by Runx/Cbfβ is likely to be independent of GATA-3. Furthermore, Runx/Cbfβ complexes bind the presumed enhancers of ILC2 functional gene loci marked by H3K27 acetylation in the IL-33-stimulated ILC2s (Figure 3). Cbfβ binding peaks overlapping with H3K27 acetylation peaks are found around the *Il5*, *Il9*, *Il13*, *Areg*, and *Nmur1* gene loci. A decrease of H3K27 tri-methylation at the *Il10* gene by the absence of Cbfb suggests that Cbfβ could repress the *Il10* locus through epigenetic mechanisms (Figure 3). Therefore, Runx/Cbfβ complexes directly contribute to the phenotypical gene expression of exhausted like ILC2s.

## 5. Conclusions

We reviewed how transcription factors regulate ILC2 differentiation and function. A new mechanism by which Runx proteins confer sustained ILC2 reactivity during allergic inflammation was identified. Exhausted-like ILC2s are a small population of over-activated ILC2s expressing inhibitory molecules. PD-1 is a good example of an inhibitory receptor that is induced on activated ILC2s and downmodulate ILC2 activity against worm infection [78]. However, PD-1 on ILC2s could be an activation marker rather than exhaustion marker, because PD-1^+^ ILC2s generally produce more T_H_2 cytokines than PD-1^-^ ILC2s. ILC2s produce IL-10 when the cells are exposed to severe allergic inflammation. However, higher T_H_2 cytokine production by IL-10-producing ILC2s suggests that IL-10-producing ILC2s are likely to be in more activated than non-IL-10 producing ILC2s [26]. We revealed that Tigit^+^IL-10^+^ ILC2s have exhausted-like features at the site of severe inflammation. Exhausted-like ILC2s are very rare in the mouse model of subacute allergy as long as Runx proteins are functional. Whether exhausted-like ILC2s are present in humans remains elusive. However, if they are present, exhausted-like ILC2s might be accumulated in patients with chronic allergy and contribute to constraining allergic responses. Thus, exhausted-like ILC2s may serve as beneficial cells to those patients and have the therapeutic potency to be designated a new target for allergic disorders. Clinical trials are warranted to clarify the physiological effects of exhausted-like ILC2s in chronic allergy.

## Abbreviations

GATA-3	GATA binding protein 3
Bcl11b	B-Cell lymphoma/leukaemia 11B
Gfi1	Growth factor independent 1 transcription repressor
RORα	RAR related orphan receptor A
Ets-1	v-ets erythroblastosis virus E26 oncogene homolog 1
Cbfb	Subunit b of core binding factor
Runx	Runt-related transcription factor
Tox	Thymocyte selection associated high mobility group box
NFIL3	Nuclear factor, interleukin 3 regulated
PLZF	Promyelocytic leukemia zinc finger
T-bet	T-box–containing protein expressed in T cells
Eomes	Eomesodermin
RORγt	RAR-related orphan receptor gamma t
AhR	Aryl hydrocarbon receptor
TCF-1	T cell factor 1
ILC	Innate lymphoid cell
Sox4	Sex determining region Y-box 4
T_H_	Helper T cell
CLP	Common lymphoid progenitor
EILP	Early innate lymphoid progenitor
CHILP	Common helper innate lymphoid progenitors
ILCP	Innate lymphoid cell precursors
Lti	Lymphoid tissue inducer
IFNγ	Interferon gamma
IL	Interleukin
GM-CSF	Granulocyte-macrophage colony-stimulating factor
TSLP	Thymic stromal lymphopoietin
TL1A	TNF superfamily ligand TL1A
Vip	Vasoactive intestinal peptide
PD-1	Programmed cell death 1
Tigit	T cell immunoreceptor with Ig and ITIM domains
ICOS	Inducible costimulator
Lag3	Lymphocyte activation gene-3
KLRG1	Killer cell lectin-like receptor G1
GITR	Glucocorticoid-induced tumor necrosis factor receptor
ICOSL	Inducible costimulatory ligand
CCL17	CC chemokine ligand 17
